# Changes in central venous saturation after major surgery, and association with outcome

**DOI:** 10.1186/cc3888

**Published:** 2005-11-08

**Authors:** Rupert Pearse, Deborah Dawson, Jayne Fawcett, Andrew Rhodes, R Michael Grounds, E David Bennett

**Affiliations:** 1 Adult Intensive Care Unit, 1st floor St James' Wing, St George's Hospital, Blackshaw Road, London SW17 0QT, UK

## Abstract

**Introduction:**

Despite recent interest in measurement of central venous oxygen saturation (ScvO_2_), there are no published data describing the pattern of ScvO_2 _changes after major general surgery or any relationship with outcome.

**Methods:**

ScvO_2 _and other biochemical, physiological and demographic data were prospectively measured for 8 hours after major surgery. Complications and deaths occurring within 28 days of enrolment were included in the data analysis. Independent predictors of complications were identified with the use of logistic regression analysis. Optimum cutoffs for ScvO_2 _were identified by receiver operator characteristic analysis.

**Results:**

Data from 118 patients was analysed; 123 morbidity episodes occurred in 64 these patients. There were 12 deaths (10.2%). The mean ± SD age was 66.8 ± 11.4 years. Twenty patients (17%) underwent emergency surgery and 77 patients (66%) were male. The mean ± SD P-POSSUM (Portsmouth Physiologic and Operative Severity Score for the enUmeration of Mortality and morbidity) score was 38.6 ± 7.7, with a predicted mortality of 16.7 ± 17.6%. After multivariate analysis, the lowest cardiac index value (odds ratio (OR) 0.58 (95% confidence intervals 0.37 to 0.9); *p *= 0.018), lowest ScvO_2 _value (OR 0.94 (0.89 to 0.98); *p *= 0.007) and P-POSSUM score (OR 1.09 (1.02 to 1.15); *p *= 0.008) were independently associated with post-operative complications. The optimal ScvO_2 _cutoff value for morbidity prediction was 64.4%. In the first hour after surgery, significant reductions in ScvO_2 _were observed, but there were no significant changes in CI or oxygen delivery index during the same period.

**Conclusion:**

Significant fluctuations in ScvO_2 _occur in the immediate post-operative period. These fluctuations are not always associated with changes in oxygen delivery, suggesting that oxygen consumption is also an important determinant of ScvO_2_. Reductions in ScvO_2 _are independently associated with post-operative complications.

## Introduction

The successful use of central venous oxygen saturation (ScvO_2_) as a haemodynamic goal in the management of early sepsis has led to interest in the use of this parameter in surgical patients [1]. ScvO_2 _measurement requires placement of a central venous catheter so that the tip lies in the superior vena cava. Readings may be taken intermittently by blood sampling and co-oximetry, or continuously with a spectrophotometric catheter. Experimental studies have shown that changes in ScvO_2 _closely reflect circulatory disturbances during periods of hypoxia, haemorrhage and subsequent resuscitation [2,3]. Fluctuations correlate well with those of mixed venous saturation (SvO_2_), although absolute values differ [2,3]. Observational studies have described changes in ScvO_2 _in various groups [4]. In particular, the prognostic significance of ScvO_2 _reductions to below 65% has been demonstrated in trauma [5], severe sepsis [6], myocardial infarction [7] and cardiac failure [8]. However, the only interventional trial of ScvO_2 _conducted so far used a goal of 70% [1].

Although the association between cardiac index (CI), oxygen delivery index (DO_2_I) and related parameters and outcome after major surgery has been well described [9-14], only limited data are available describing ScvO_2 _values in the peri-operative period [15]. The physiology of ScvO_2 _disturbances is complex. The value of ScvO_2 _is determined by changes in oxygen delivery and consumption, both of which are subject to considerable variation during the peri-operative period [4]. It is not appropriate to assume that either the normal value or fluctuations in ScvO_2 _will be similar to those of other patient groups. If ScvO_2 _is to be used in the haemodynamic assessment of surgical patients, more detailed information is required describing fluctuations during the peri-operative period. The aim of this study was to describe changes in ScvO_2 _after major general surgery and their relationship to outcome.

## Methods

### Patients

ScvO_2 _data were collected from adult patients enrolled in the randomised study of post-operative goal-directed therapy (GDT) [16]. All patients were deemed to be at high risk of post-operative complications and were admitted to the intensive care unit (ICU) immediately after major surgery. This study was approved by the Local Research Ethics Committee of St George's Healthcare National Health Service Trust.

### Assessment

All patients had arterial and central venous catheters placed before the commencement of surgery. The central venous catheter was positioned with the tip within the superior vena cava immediately above the right atrium. This position was verified by chest radiograph and adjusted if necessary. The following parameters were monitored continuously from arrival in the ICU immediately after surgery and for the next 8 hours: electrocardiograph, pulse oximetry, invasive arterial pressure, central venous pressure and cardiac output. Arterial and central venous blood gas analyses were performed by intermittent blood sampling and co-oximetry (ABL 700; Radiometer, Copenhagen, Denmark) at baseline and hourly during the 8 hours after surgery. This equipment was calibrated each hour, and routine quality control checks were performed. Cardiac output was measured by lithium indicator dilution and pulse power analysis (LiDCO plus system; LiDCO Ltd., Cambridge, UK). P-POSSUM (Portsmouth Physiologic and Operative Severity Score for the enUmeration of Mortality and morbidity) and APACHE II (Acute Physiology and Chronic Health Evaluation II) scores were calculated at admission to the ICU [17,18]. Complications and deaths occurring within 28 days of enrolment were included in the data analysis. Complications were prospectively defined, diagnosed by clinical staff and verified by a member of the research team. This process involved daily inspection of notes, radiological investigations, laboratory data and clinical assessment.

### Clinical management

Protocols for cardiovascular management during the immediate post-operative period are provided in detail elsewhere [16]. Fluid challenges were guided by central venous pressure in 56 patients and by stroke volume in 61 patients. The latter group also received dopexamine if they did not achieve a DO_2_I of 600 ml min^-1 ^m^-2 ^with fluid alone (GDT group). Once the 8-hour study period was complete, all patients received standard care for the remainder of their ICU and hospital stay. ScvO_2 _data were not used to guide clinical management at any stage.

### Statistical analysis

Data are presented as means ± SD where normally distributed, as medians (interquartile range) where not normally distributed or, for categorical variables, as a percentage of the group from which they were derived. Normality was tested with the Kolmogorov–Smirnov test. Categorical data were tested with Fisher's exact test. Continuous data were tested with the *t *test where normally distributed and the Mann–Whitney *U *test where not normally distributed. Trends in physiological parameters over time in the two groups were compared with repeated-measures analysis of variance with Tukey's correction for multiple comparisons.

Univariate analysis was performed to test association with complications and death. For data recorded hourly during the study period, the baseline values, lowest values and the mean over the 8-hour study period were tested. A multiple logistic regression model was used to identify independent risk factors for post-operative complications. A stepwise approach was used to enter new terms into the logistic regression model, where *p *< 0.05 was set as the limit for inclusion of new terms. Results of logistic regression are reported as adjusted odds ratios (ORs) with 95% confidence intervals. Receiver operator characteristic curves were constructed to identify optimal cutoff values for association with outcome. The optimum cutoff was defined as the value associated with the highest sum of sensitivity and specificity. Analysis was performed with GraphPad Prism version 4.0 for Windows (GraphPad Software, San Diego, CA, USA) and significance was set at *p *< 0.05.

## Results

Data was collected from 117 patients between November 2002 and August 2004. Five patients were excluded from the analysis because ScvO_2 _data were collected with a spectrophotometric catheter. Sixty-four patients developed 123 complications in all. There were 12 deaths (10.2%). The mean ± SD age was 66.8 ± 11.4 years. Twenty patients (17%) underwent emergency surgery and 77 patients (66%) were male. The APACHE II score was 9.5 ± 4.1, with a predicted mortality of 10.3 ± 9.0%. The P-POSSUM score was 38.6 ± 7.7, with a predicted mortality of 16.7 ± 17.6%. Fifty-seven (49%) patients were extubated within 1 hour of surgery and a further 29 (25%) were extubated before the end of the 8-hour study period.

### Associations with outcome

Commonly measured physiological, biochemical and demographic variables are presented in Tables 1 and 2. Although derangements in CI, DO_2_I and ScvO_2 _were frequently observed, other parameters remained within the normal range or were only slightly abnormal. Univariate analysis identified five variables associated with post-operative complications. These were the lowest ScvO_2 _value, the lowest DO_2_I value, the lowest CI value, the P-POSSUM score and the use of GDT. After multivariate analysis, the lowest CI value (OR 0.58 (95% confidence interval 0.37 to 0.9); *p *= 0.018), the lowest ScvO_2 _value (OR 0.94 (0.89 to 0.98); *p *= 0.007) and P-POSSUM score (OR 1.09 (1.02 to 1.15); *p *= 0.008) were independently associated with post-operative complications. The lowest DO_2_I value and use of GDT were not independent predictors of outcome. The optimal value of ScvO_2 _to discriminate between patients who did or did not develop complications was 64.4% (sensitivity 67%, specificity 56%). Univariate analysis identified no associations with mortality.

### Trends in ScvO_2_

**Figure 1:**
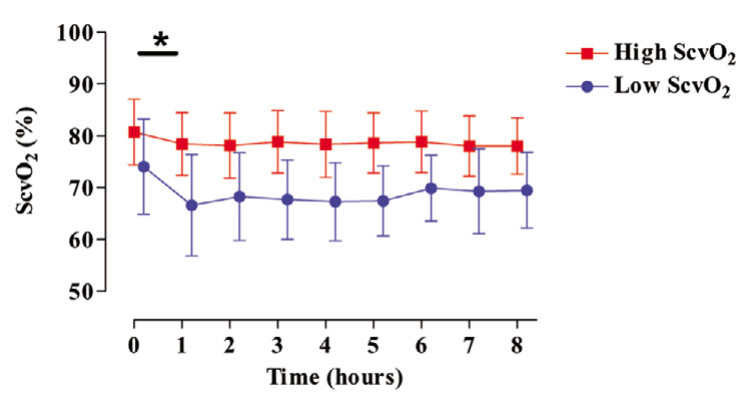
Central venous saturation (ScvO_2_) in the 8 hours after major surgery. Results are means ± SD. *p < 0.0001 for low ScvO_2 _group; *p *= 0.02 for high ScvO_2 _group. The difference between the high and low groups is significant overall and for each individual time point (*p *< 0.0001).

**Figure 2:**
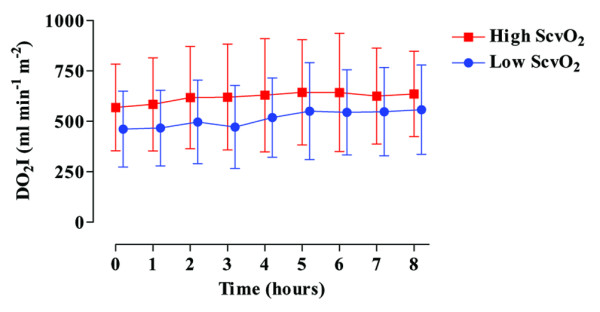
Oxygen delivery index (DO_2_I) in the 8 hours after major surgery. Results are means ± SD. The difference between the group with high central venous saturation (ScvO_2_) and the low ScvO_2 _group is significant overall (*p *= 0.005) but not for individual time points 7 and 8.

Patients were divided into two groups by using the optimal cutoff value for ScvO_2_. Those in whom the lowest ScvO_2 _value was 64.4% or below were defined as the low ScvO_2 _group and those in whom the lowest value was above 64.4% were defined as the high ScvO_2 _group (see Table 3). Trends in ScvO_2 _and DO_2_I are presented in Figures 1 and 2. During the first post-operative hour there was a significant decrease in ScvO_2 _in both the high ScvO_2 _group (79.8 ± 6.3% to 77.7 ± 5.8%; *p *= 0.016) and the low ScvO_2 _group (74.6 ± 9.7% to 66.6 ± 10.3%; *p *< 0.0001). DO_2_I and CI values did not change significantly during this time.

## Discussion

The major finding of this study is the occurrence of considerable fluctuations in ScvO_2 _after major general surgery that have prognostic significance. Multivariate analysis identified the lowest ScvO_2 _value, lowest CI value and P-POSSUM score as independent predictors of complications. This observation supports the hypothesis that the association between reductions in ScvO_2 _and outcome is similar to that observed previously for CI and DO_2_I [9-13]. It is interesting to note that P-POSSUM score was an independent predictor of complications, but APACHE II score was not. This may be because P-POSSUM score was designed for use in surgical patients using data from the UK, whereas APACHE II was designed for use in mixed groups of critically ill patients using data from North America [17,18]. As might be expected, the use of GDT was associated with fewer post-operative complications. However, this association was not independent of other predictors of outcome. The observation of collinearity between CI, DO_2_I and the use of GDT suggests that the level of DO_2_I achieved by individual patients is more important than the approach to haemodynamic management.

The optimal cutoff value of ScvO_2 _for prediction of complications was 64.4%. This is very similar to the value (65%) identified in other patient groups [5-7]. Large fluctuations in ScvO_2 _occur during the peri-operative period. Values of ScvO_2 _decreased significantly during the first hour after surgery, while CI and DO_2_I remained unchanged. A significant increase in oxygen consumption therefore occurred during this period despite the fact that fewer than half of the patients were extubated within 1 hour of surgery. This finding is consistent with previous findings in cardiac surgical patients [14], as well as earlier work by Shoemaker [13]. Post-operative oxygen consumption is determined by various factors including pain, emergence from anaesthesia, body temperature and shivering. Peri-operative disturbances of ScvO_2 _cannot therefore be assumed to relate solely to DO_2_I.

The 8-hour mean of ScvO_2 _was 75.0% in patients who did not develop post-operative complications. This value was comparable to previous measurements in healthy conscious patients [19,20], but higher than those taken immediately before induction of anaesthesia [15] and in patients with good outcome after trauma, severe sepsis, cardiac failure or myocardial infarction [5-8]. It is notable that derangements in CI, DO_2_I and ScvO_2 _were observed in the absence of similar disturbances in other commonly measured biochemical and physiological variables. This was despite the high rates of morbidity and mortality in the study population. It is possible that disturbances in ScvO_2_, CI and DO_2_I might indicate the presence of occult tissue hypoperfusion before disturbances in other parameters.

The use of observational data from an interventional trial has both advantages and disadvantages. In this study, goals for arterial oxygen saturation, haemoglobin, heart rate, mean arterial pressure, serum lactate and urine output were the same in all patients. All clinical management and data collection were closely supervised by a member of the research team in accordance with a carefully defined treatment protocol. The benefit of such rigorous study design must be offset against the fact that, in some patients, intravenous fluid administration was guided by central venous pressure, whereas in others fluid management was guided by stroke volume and supplemented with low-dose dopexamine. It is an inherent problem with studies of this type that the predictive nature of certain variables may relate both to the initial cardiovascular disturbance and subsequent attempts to correct it. The large number of statistical comparisons performed in the univariate analyses may seem speculative. This is not the case; comparisons made were of variables in which an association with outcome had previously been suggested [9-14,17,18,21]. We were therefore obliged to identify all such associations in the available data.

## Conclusion

Reductions in ScvO_2 _are common after major surgery and are associated with an increased rate of post-operative complications. Peri-operative changes in ScvO_2 _relate to both oxygen consumption and delivery. Further evaluation of peri-operative trends in ScvO_2 _should be performed before this variable is used as a haemodynamic goal in surgical patients.

## Key messages

• 	The successful use of central venous saturation in the management of severe sepsis has led to interest in the use of this variable in surgical patients.

• 	This analysis suggests that central venous saturation may have prognostic significance following major surgery.

• 	Further evaluation of peri-operative trends in central venous saturation is required.

## Abbreviations

APACHE = Acute Physiology and Chronic Health Evaluation; CI = cardiac index; DO_2_I = oxygen delivery index; GDT = goal-directed therapy; ICU = intensive care unit; OR = odds ratio; P-POSSUM = Portsmouth Physiologic and Operative Severity Score for the enUmeration of Mortality and morbidity; ScvO_2 _= central venous saturation.

## Competing interests

RP received a travel grant from LiDCO Ltd. to present data at an international meeting. JF has previously performed consultancy work for LiDCO Ltd. DB currently performs consultancy work for LiDCO Ltd. and has previously performed consultancy work for Deltex Ltd. No other competing interests are declared.

## Authors' contributions

RP, AR and DB were responsible for study design. RP, DD and JF were responsible for data collection. All authors were involved in data analysis and drafting the manuscript and approved the final version. All authors had full access to data and take responsibility for the integrity of the data and the accuracy of the analysis.

## Figures and Tables

**Table 1:** Demographic and biochemical data for patients with and without post-operative morbidity

Data class	Complications (*n *= 64)	No complications (*n *= 53)	*p*
Demographic			
Age (years)	67.0 ± 12.3	66.7 ± 10.4	0.89
Blood loss (ml)	1,200 (520–2,000)	1,000 (725–2,350)	0.88
GDT	27/64 (42%)	35/53 (66%)	0.02*
APACHE II score	9.9 ± 4.3	9.0 ± 3.8	0.28
P-POSSUM score	40.1 ± 8.1	36.8 ± 6.8	0.02*
ASA score	3 (2–3)	3 (2–3)	0.61
Biochemical			
Baseline base excess (mmol l^-1^)	-2.62 ± 3.03	-2.40 ± 3.31	0.71
Lowest base excess (mmol l^-1^)	-3.67 ± 3.04	-4.30 ± 3.17	0.29
Base excess, 8-hour mean (mmol l^-1^)	-2.73 ± 3.83	-2.31 ± 3.15	0.44
Baseline lactate (mmol l^-1^)	1.49 ± 0.81	1.38 ± 0.74	0.43
Highest lactate (mmol l^-1^)	1.93 ± 1.30	1.80 ± 0.88	0.55
Lactate, 8-hour mean (mmol l^-1^)	1.29 ± 0.81	1.23 ± 0.55	0.65

*Statistically significant difference. Data are presented as means ± SD, medians (interquartile range) or absolute values (%). APACHE, Acute Physiology and Chronic Health Evaluation; GDT, patients receiving goal-directed therapy; P-POSSUM, Portsmouth Physiologic and Operative Severity Score for the enUmeration of Mortality and morbidity

**Table 2:** Haemodynamic data for patients with and without post-operative morbidity

Data class	Complications (*n *= 64)	No complications (*n *= 53)	*p*
Haemodynamic			
Baseline heart rate (beats min^-1^)	82.1 ± 21.4	81.5 ± 15.5	0.87
Highest heart rate (beats min^-1^)	100.3 ± 19.9	106.6 ± 22.4	0.11
Heart rate, 8-hour mean (beats min^-1^)	86.0 ± 16.3	90.3 ± 16.0	0.15
Baseline MAP (mmHg)	93.9 ± 19.4	99.6 ± 20.2	0.12
Lowest MAP (mmHg)	74.5 ± 14.7	76.3 ± 12.8	0.48
MAP, 8 hour mean (mmHg)	90.8 ± 15.3	92.5 ± 12.9	0.52
Baseline CI (l min^-1 ^m^-2^)	3.59 ± 1.39	3.87 ± 1.43	0.30
Lowest CI (l min^-1 ^m^-2^)	2.74 ± 0.79	3.25 ± 1.32	0.02*
CI, 8-hour mean (l min^-1 ^m^-2^)	3.93 ± 1.07	4.20 ± 1.55	0.30
Baseline DO_2_I (ml min^-1 ^m^-2^)	494 ± 191	541 ± 229	0.26
Lowest DO_2_I (ml min^-1 ^m^-2^)	364 ± 158	445 ± 218	0.02*
DO_2_I, 8-hour mean (ml min^-1 ^m^-2^)	517 ± 206	581 ± 255	0.13
Baseline stroke volume (ml)	84 ± 30	88 ± 33	0.44
Lowest stroke volume (ml)	62 ± 25	70 ± 30	0.12
Stroke volume, 8-hour mean (ml)	70 ± 31	86 ± 32	0.29
ScvO_2_			
Baseline (%)	76.2 ± 9.9	78.7 ± 6.2	0.11
Lowest (%)	63.4 ± 10.4	67.1 ± 7.7	0.03*
8-hour mean (%)	73.0 ± 6.6	75.0 ± 5.6	0.09

*Statistically significant difference. Data are presented as means ± SD. CI, cardiac index; DO_2_I, oxygen delivery index; MAP, mean arterial pressure; ScvO_2_, central venous saturation.

**Table 3:** Demographic and outcome data for high-ScvO_2 _and low-ScvO_2 _groups

Parameter	High ScvO_2_	Low ScvO_2_	*p*
Number in group	64	53	-
Age	66 ± 12	69 ± 11	0.40
P-POSSUM	37.8 ± 7.4	39.0 ± 7.5	0.39
APACHE II score	9.8 ± 4.3	9.0 ± 3.9	0.30
Length of hospital stay (days)	12 (9–15)	14 (9–25)	0.25
Complications (number of patients)	29 (45%)	35 (66%)	0.03*
Complications (episodes per patient)	0.8 ± 1.1	1.4 ± 1.4	0.04*
Mortality	7 (11%)	4 (7%)	0.54

*Statistically significant difference. Data are presented as means ± SD, medians (interquartile range) or absolute values (%). APACHE, Acute Physiology and Chronic Health Evaluation; P-POSSUM, Portsmouth Physiologic and Operative Severity Score for the enUmeration of Mortality and morbidity; ScvO_2_, central venous saturation.
